# Contrast-enhanced ultrasonography–CT/MRI fusion guidance for percutaneous ablation of inconspicuous, small liver tumors: improving feasibility and therapeutic outcome

**DOI:** 10.1186/s40644-023-00650-y

**Published:** 2024-01-03

**Authors:** Yuna Lee, Jeong Hee Yoon, Seungchul Han, Ijin Joo, Jeong Min Lee

**Affiliations:** 1 Department of Radiology, Seoul National University Hospital, #101 Daehak-ro, Jongno-gu, Seoul, 03080 Korea; 2 Department of Radiology, Seoul National University College of Medicine, Seoul, Korea; 3 Institute of Radiation Medicine, Seoul National University Medical Research Center, Seoul, Korea

**Keywords:** Liver malignancies, Fusion imaging, Ultrasound, Ablation, Treatment outcome

## Abstract

**Background:**

Percutaneous radiofrequency ablation (RFA) is pivotal for treating small malignant liver tumors, but tumors often remain inconspicuous on B-mode ultrasound (US). This study evaluates the potential of CEUS-CT/MRI fusion imaging (FI) to improve tumor visibility and the associated RFA outcomes for small (≤ 3 cm) malignant liver tumors that were inconspicuous on US.

**Methods:**

Between January 2019 and April 2021, a prospective study enrolled 248 patients with liver malignancies (≤ 3 cm) that were poorly visible on B-mode US. Tumor visibility and ablation feasibility were assessed using B-mode US, US-CT/MRI FI, and CEUS-CT/MRI FI, and graded on a 4-point scale. CEUS was employed post-registration of US and CT/MRI images, utilizing either SonoVue or Sonazoid. Comparisons between US-based and CEUS-based fusion visibility and feasibility scores were undertaken using the Friedman test. Moreover, rates of technical success, technique efficacy, local tumor progression (LTP), and major complications were assessed.

**Results:**

The cohort included 223 hepatocellular carcinomas (HCCs) (89.9%) and 23 metastases (9.3%), with an average tumor size of 1.6 cm. CEUS-CT/MRI FI demonstrated a significant advantage in tumor visibility (3.4 ± 0.7 vs. 1.9 ± 0.6, P < 0.001) and technical feasibility (3.6 ± 0.6 vs. 2.9 ± 0.8, P < 0.001) compared to US-FI. In 85.5% of patients, CEUS addition to US-FI ameliorated tumor visibility. Technical success was achieved in 99.6% of cases. No severe complications were reported. One and two-year post CEUS-CT/MRI FI-guided RFA estimates for LTP were 9.3% and 10.9%, respectively.

**Conclusions:**

CEUS-CT/MRI FI significantly improves the visualization of tumors not discernible on B-mode US, thus augmenting percutaneous RFA success and delivering improved therapeutic outcomes.

**Trial registration:**

ClinicalTrials.gov, NCT05445973. Registered 17 June 2022 – Retrospectively registered, http://clinicaltrials.gov/study/NCT05445973?id=NCT05445973&rank=1.

**Supplementary Information:**

The online version contains supplementary material available at 10.1186/s40644-023-00650-y.

## Background

Percutaneous radiofrequency ablation (RFA) has been widely implemented as a curative treatment for liver malignancies [1, 2]. The overall survival post-RFA is comparable to that of surgical outcomes for small hepatocellular carcinomas (HCCs) or colorectal metastases (≤ 3 cm) [3–5]. US guidance for RFA is common in Asia because the real-time capability of ultrasound (US) allows precise electrode placement [1, 6, 7]. However, precisely targeting small liver malignancies with poor sonographic conspicuity in US-guided RFA is often challenging [8]. According to previous studies [1, 2], feasibility rates were reported in a range of 27%~55%, and the invisibility of index tumor on US or the absence of safe access routes were the two most common reasons for infeasibility. To solve this problem, CEUS [7, 9–11] or real-time fusion imaging (FI) of US and CT/MRI have been used [12–14]. Both approaches have been reported to improve the confidence for tumor localization in RFA for small HCCs with poor B-mode US visibility [7, 9, 10, 12–15]. However, when an index tumor is entirely invisible on B-mode US, neither method is ideal, especially for multiple-electrode RFA, due to inherent registration errors on FI [13, 16, 17] or insonation-induced bubble destruction on CEUS with SonoVue [7]. Therefore, there is a clinical need to compensate for the inherent registration errors of US-CT/MRI FI for inconspicuous tumors on both US and US-CT/MRI FI.

Recent studies reported that after adding CEUS using Sonazoid (GE Healthcare, Waukesha, WI, USA) or SonoVue to FI, 83.3–90% of target liver cancers that were initially difficult to visualize on FI became conspicuous, allowing them to be directly targeted for RFA [16, 18, 19]. However, these studies had limitations, including a retrospective study design, a small sample size (< 30 inconspicuous tumors), or a short-term follow-up (< 1 year). Furthermore, no prospective study has explored the value of CEUS-CT/MRI FI, using both SonoVue and Sonazoid as contrast agents, for localizing index tumors and guiding RFA procedures in patients with inconspicuous tumors on B-mode US and reported its therapeutic impact on local tumor control.

Therefore, the present study aimed to evaluate the potential of CEUS-CT/MRI fusion imaging (FI) in enhancing the visibility of tumors and associated RFA outcomes for patients with small malignancies that are inconspicuous on B-mode US.

## Methods

The institutional review board approved this prospective study (IRB No 1811-136-989). All patients provided written informed consent prior to enrollment. The primary endpoints were the tumor visibility score, and the technical feasibility score for RFA using CEUS-CT/MRI FI scored on a 4-point scale. The secondary endpoints were technical success and technique efficacy, local tumor progression (LTP) rates, and major complications after CEUS-CT/MRI FI–guided RFA. Financial support was provided by Canon Medical (No. 0620101950) and Siemens Healthineers (No. 0620200760). All authors have complete control over all information and data submitted for publication with patients’ consent.

### Participants

Between January 2019 and April 2021, patients referred to our institution’s radiology department for RFA for enrollment in this prospective study were screened. Before the main RFA procedure, tumor visibility was scored by an attending abdominal radiologist (J.M.L., 20 years of clinical experience with RFA) in an interventional US suite with a clinical fellow or a senior resident on a 4-point scale. Tumors with a score of 1 or 2 were regarded as inconspicuous (Supplementary Table 1).

The following inclusion criteria were applied: (1) inconspicuous or invisible index tumors for ablation on US; (2) tumor size ≤ 3 cm; (3) pathologic diagnosis of primary or secondary liver malignancy or imaging-based diagnosis of HCC according to the American Association for the Study of Liver Disease guidelines [20] or viable HCC according to the Liver Imaging Reporting and Data System treatment response algorithm [21]; (4) consideration of curative-intent RFA. The exclusion criteria for our study included: (1) tumors that were well-visible (with a visibility score of 3 or 4) on the planning B-mode Ultrasound; (2) absence of available data from multiphase CT or MRI performed in the 3-month period preceding the procedure; (3) poor quality registration of US-CT/MRI fusion imaging; (4) RFA (Radiofrequency Ablation) planned with palliative intent; (5) any therapeutic procedures conducted between the last CT or MRI examination and the ablation procedure; and (6) contraindications for the conventional RFA procedure as per the guidelines of our institution, which are uncontrollable coagulopathy (platelet count < 50,000/µL or international standard ratio ≥ 1.6), a low level of cooperation, difficulty in sedation, portal vein thrombus, the tumor abutting the portal vein, or larger bile ducts than the segmental branches. Poor-quality registration of US-FI was defined as poor matching of segmental branches of the portal vein and hepatic vein of the segment bearing the target tumor between two imaging sets, as well as deformation of the liver capsule [14, 22].

All patients had available contrast-enhanced liver CT and/or Gd-EOB-DTPA-enhanced MRI within 3 months before ablation. All patient underwent B-mode US and US-CT/MRI FI examinations to assess the size and number of tumors, as well as the anatomical relationship of tumors with neighboring vital structures [12].

### US, fusion imaging, and CEUS-fusion imaging during the RFA planning session

After planning US, real-time image fusion between US and CT/MRI (US-FI) was processed using commercially available US-CT/MRI FI techniques and ultrasound scanners (RS 85, Samsung Medison; Apolio i-800, Canon Medical Systems; Acuson Sequoia, Siemens Healthineers). The equipment contained a position sensing unit with two electromagnetic sensors, as well as a magnetic field generator. The set of CT or MRI images that portrayed the target tumor most clearly was chosen for FI, and rigid registration techniques were used [22]. Previous studies have described the fusion process between US images and CT/MRI [22, 23]. The operator and a clinical fellow or a senior resident assessed the target tumor visibility, tumor localization confidence, the safety of the access route, and the expected.

Next, CEUS with either SonoVue (Sulfer Hexafluoride, Bracco, Milan, Italy) or Sonazoid (Perflubutane, GE Healthcare, Waukesha, WI, USA) was performed with US-FI to confirm the target tumor’s location. There were no specific selection criteria for SonoVue or Sonazoid, but SonoVue was used in the first half of the study period, and Sonazoid was used in the latter half due to their availability at our institution. Intravenous injections of SonoVue were performed at a 2.4-mL dose, while Sonazoid was administered through intravenous injections with a 0.015-mL/kg dose, followed by a 10-mL normal saline flush. CEUS images were obtained during the arterial phase (10–40 s post-contrast injection), portal phase (60–90 s), and delayed phase (3 min). When Sonazoid was used, the Kupffer phase (more than 10 min) was additionally obtained. The technical details of CEUS were described in previous studies [24]. The operator and a clinical fellow or a senior resident reassessed target tumor visibility, tumor localization confidence, the safety of the access route, and the expected technical feasibility using the scoring system mentioned above.

The tumors were grouped as follows: group A, clearly visualized on both US-FI and CEUS-CT/MRI FI; group B, poorly visualized on US-FI but clearly visualized on CEUS-CT/MRI FI; and group C, poorly visualized on both US-FI and CEUS-CT/MRI FI (Supplementary Table 1). The definition of “clearly visible” was a visibility score of 3–4 (visible tumor with partial delineation of tumor margin or clearly visible), while the definition of “poorly visualized” was a target tumor visibility score of 1 or 2 (definitely invisible or subtle visualization). The scoring system is presented in greater detail in Supplementary Table 1.

### RFA procedure

A single senior radiologist (J.M.L., 20 years of experience in RFA) conducted all percutaneous RFA procedures in the US suite, with assistance from a clinical fellow or a senior resident. The ablation procedure has been described elsewhere [23]. In brief, ablation was performed using a 200-W multichannel generator (VIVA RF System, STARmed) and a switching monopolar technique with separate clustered electrodes (Octopus electrodes; STARmed, Goyang, Kyunggi, Korea) [25, 26]. Tumor targeting was done under the guidance of CEUS-CT/MRI FI, and the ablation procedure was monitored with US-FI. The ablation procedure was terminated when the operator expected to complete the ablation of the index tumor with a minimum 5-mm ablative margin on the US-FI images [15].

An immediate post-procedural quadriphasic liver CT scan was obtained to evaluate the ablation margin and possible complications using the CT unit (Discovery CT750HD; GE Healthcare, Waukesha, WI, USA) located next to the RFA unit. Contrast medium (1.35 mL/kg of Ultravist 370; Bayer Healthcare) was administered intravenously at 2.0 to 4.0 mL/s using a power injector (Multilevel CT; Medrad, Indianola, PA, USA), followed by a 30–40-mL saline flush. Technical success was defined as complete coverage of the tumor by the ablation zone with an ablation margin on immediate follow-up imaging, according to the recommendation of the International Working Group on Image-Guided Tumor Ablation [2]. Major complications were defined as those resulting in substantial morbidity and disability [2].

### Post-procedural follow-up

One month after procedure, follow-up contrast-enhanced CT or MRI was performed; thereafter, serial CT or MRI was conducted at 3-month intervals during the first year post-procedure and at 4- to 6-month intervals in the second year. The determination of technique efficacy was made based on multiphasic liver CT or MRI performed one-month post-procedure, in accordance with previous research [27]. Throughout follow-up, LTP (the appearance of a new tumor focus along the margin of the ablation zone) was assessed [15].

### Statistical analysis

The Shapiro-Wilk test was used to assess whether the data showed a normal distribution. The significant difference in categorical variables was determined using the chi-square or Fisher exact test. The Friedman test was used to compare pre-procedural parameters, such as tumor visibility and expected technical feasibility scores. Kaplan-Meier analysis and the log-rank test were used to estimate and compare cumulative LTP rates. All statistical analyses were conducted using SPSS version 25 (IBM Corp., Armonk, NY, USA), and the threshold for statistical significance was a P-value of < 0.05.

## Results

### Characteristics of patients and tumors

**Fig. 1:**
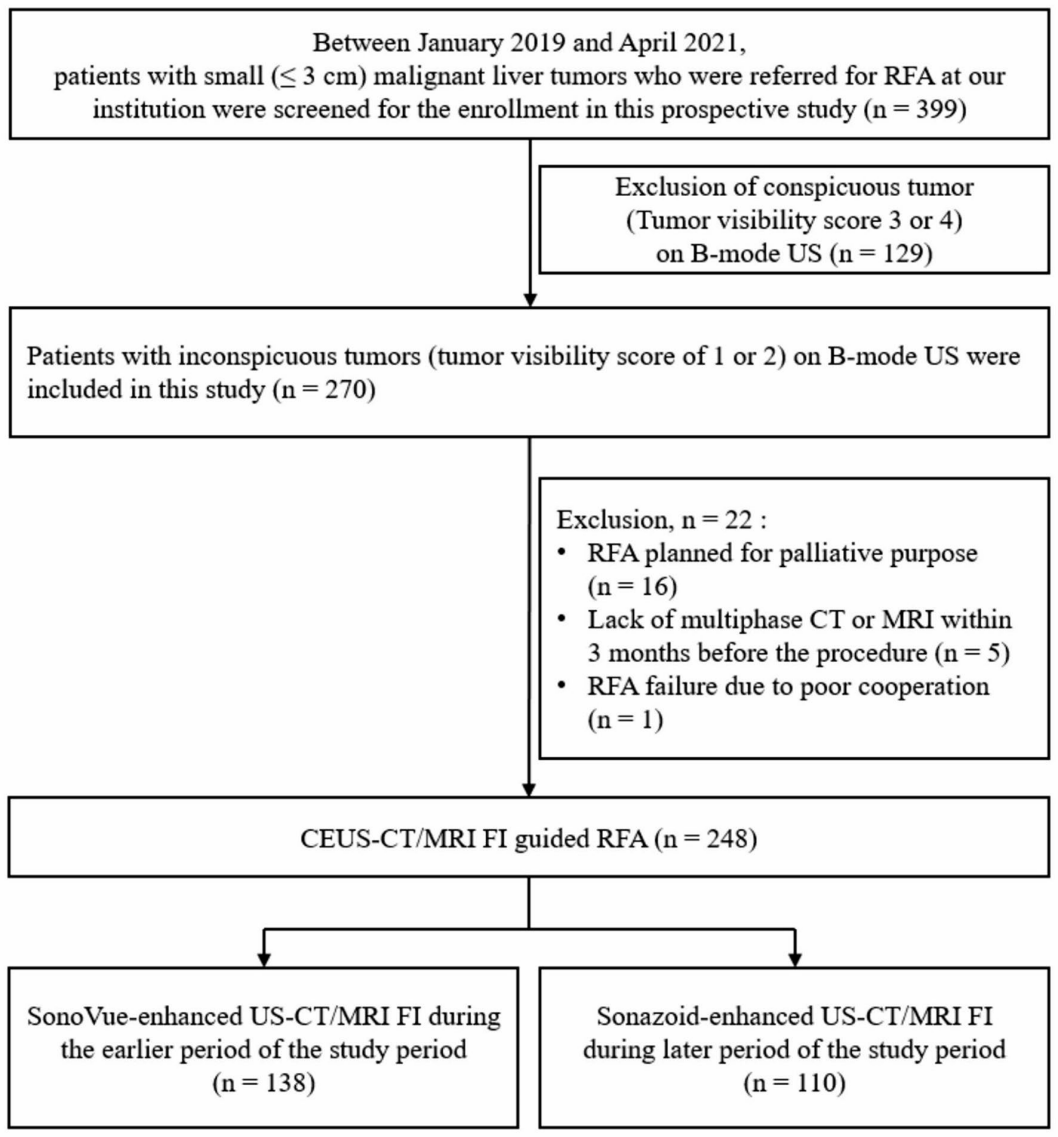
Flowchart presenting the process of patient enrollment

Between January 2019 and April 2021, 399 patients with inconspicuous, small (≤ 3 cm) who were referred to the radiology department for RFA were screened. One hundred fifty-one candidates failed to meet the eligibility criteria, and finally, 248 participants (male = 187) with 248 malignant liver tumors were enrolled (Fig. 1). Two hundred twenty-three patients had HCCs, 23 had metastases, and 2 had IHCCs. All patients underwent percutaneous RFA under CEUS- FI guidance to treat liver tumors and were included in the evaluation of therapeutic outcomes. For CEUS, SonoVue was used in 138 patients during the first half of the study period, and Sonazoid was used in 110 patients during the second half.

Patients’ baseline characteristics are summarized in Table 1. There were 223 HCCs (89.9%, 223/248), 23 metastases (23/248, 9.3%), and two IHCCs (0.8%, 2/248). The mean tumor size was 1.6 ± 0.6 cm. Recurrent tumors were present in 73% of cases (181/248) and de novo tumors in 27% (67/248). The SonoVue and Sonazoid groups showed no significant differences in tumor size, the percentage of recurrent tumors, and Barcelona Clinic Liver Cancer stage (Table 2). The median follow-up period was 14.6 months for patients included in the LTP and survival analyses.

**Table 1:** Baseline characteristics of study population

Baseline characteristics (n = 248 patients, 248 tumors)	
	Total
Sex (M: F)	187:61 (75.4:24.6)
Age (year, mean ± SD)	66.2 ± 8.4
Child-Pugh classification (A/B)	241/7
BCLC stage*	
Very early (0)	159/224 (71.0)
Early (A)	61/224 (27.2)
Intermediate (B)	4/224 (1.8)
**Image modality used for fusion process**	
CT	36/248 (14.5)
MRI	212/248 (85.5)
Median interval between imaging and procedure (days, range)	24 [0–63]
**Tumor location**	
Left lobe (S2, S3, S4)	62/248 (25.0)
Right superior segments (S7, S8)	99/248 (40.0)
Right inferior segments (S5, S6)	87/248 (35.1)
Tumor size (cm, mean ± SD)	1.6 ± 0.6
< 2 cm	168/248 (67.7)
≥ 2 cm, < 2.5 cm	64/248 (25.8)
≥ 2.5 cm	16/248 (6.5)
**Tumor diagnosis**	
HCC	223/248 (89.9)
Liver metastasis	23/248 (9.3)
Other malignancies	2/248 (0.8)
**Tumor nature**	
De novo tumor	67/248 (27.0)
Recurred tumor	181/248 (73.0)
**Underlying liver disease**	
Alcoholic liver disease	8/248 (3.2)
HBV	177/248 (71.4)
HCV	21/248 (8.5)
Others	42/248 (16.9)
**Previous treatment for HCC**	
RFA	114/248 (46.0)
TACE	110/248 (44.4)
PEIT	16/248 (6.5)
Surgical resection	51/248 (20.6)
Insertion of artificial ascites	212/248 (85.5)
Major complications**	0/248 (0)
Median follow up period (months, range)	14.6 [0-31.3]
Mean procedure time (minutes, range)	7.8 [2-22.5]

*Note*: Unless indicated, data are number of patients or tumors, with the percentage in parentheses. *Statistics for only HCCs, otherwise for liver tumors, **Includes vascular or bile duct injury, massive bleeding, and pneumothorax. BCLC stage = Barcelona clinic liver cancer stage

**Table 2:** Baseline characteristics for Sonovue group and Sonazoid group

Baseline characteristics for SonoVue (n = 138 tumors) and Sonazoid (n = 110 tumors)			
	SonoVue	Sonazoid	P value
Sex (M: F)	105:33 (76.1:23.9)	82:28 (74.5:25.5)	0.779
Age (year, mean ± SD)	66.1 ± 8.2	66.3 ± 8.7	0.563
Child-Pugh classification (A/B)	133/5	108/2	0.394
BCLC stage*			
Very early (0)	95/130 (73.1)	64/94 (68.1)	0.494
Early (A)	32/130 (24.6)	29/94 (30.9)	0.301
Intermediate (B)	3/130 (2.3)	1/94 (1.1)	0.488
**Tumor location**			
Left lobe (S2, S3, S4)	34/138 (24.6)	28/110 (25.5)	0.883
Right superior segments (S7, S8)	54/138 (39.1)	45/110 (40.9)	0.776
Right inferior segments (S5, S6)	50/138 36.2)	37/110 (33.6)	0.670
Tumor size (cm, mean ± SD)	1.5 ± 0.5	1.7 ± 0.6	0.056
**Tumor diagnosis**			
HCC	129/138 (93.5)	94/110 (85.5)	0.054
Liver metastasis	7/138 (5.1)	16/110 (14.5)	0.014
Other malignancies	2/138 (1.4)	0/110 (0)	0.504
**Tumor nature**			0.128
De novo tumor	32/138 (23.2)	35/110 (31.8)	
Recurred tumor	106/138 (76.8)	75/110 (68.2)	
**Underlying liver disease**			
Alcoholic liver disease	5/138 (3.6)	3/110 (2.7)	0.692
HBV	102/138 (73.9)	75/110 (68.2)	0.416
HCV	14/138 (10.1)	7/110 (6.4)	0.477
Others	17/138 (12.3)	25/110 (22.7)	0.045
Insertion of artificial ascites	121/137 (87.7)	91/110 (82.7)	0.271
Median follow up period (months, range)	27.5 [0.7–31.3]	14.9 [0-15.9]	
Mean procedure time (minutes, range)	6.8 [2-16.8]	9.0 [2-22.5]	

*Note*: Unless indicated, data are number of patients or tumors, with the percentage in parentheses. * Statistics for only HCCs, otherwise for liver tumors

BCLC stage = Barcelona clinic liver cancer stage

### Tumor visibility on US, US-FI, and CEUS-CT/MRI FI

US-CT/MRI FI and CEUS-CT/MRI FI improved visibility and increased localization confidence in 38 tumors (15.3%, 38/248) (group A). In addition, 183 tumors (73.8%, 183/248) showed poor visibility on US-FI but improved visibility and localization confidence with CEUS-CT/MRI FI, which, therefore, aided in ablation procedures that would have otherwise been technically difficult with only B-mode US or US-FI guidance (group B). However, 27 tumors (10.9%, 27/248) were inconspicuous on both US-FI and CEUS-CT/MRI FI (group C). Among the group A tumors, CEUS-CT/MRI FI improved the visibility score from 3 to 4 in 29 tumors (76.3%, 29/38). Therefore, the addition of CEUS to US-FI provided value by improving tumor localization confidence in 85.5% (212/248) of the study patients compared with US or US-FI.

### Comparison of parameters before and after CEUS in the RFA planning session

**Fig. 2:**
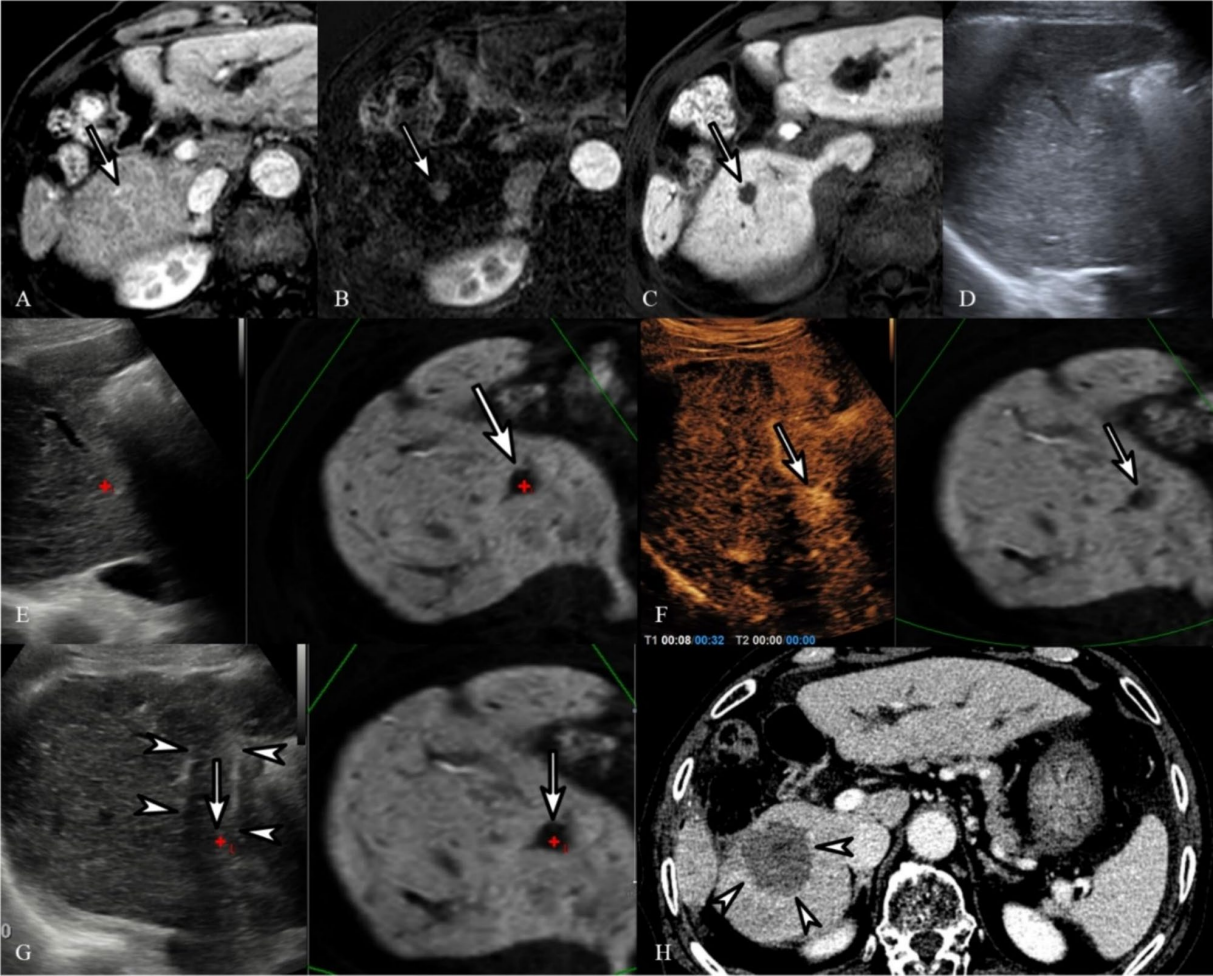
Images of using SonoVue in radiofrequency ablation. Images of a 76-year-old man who had a 1.5-cm hepatocellular carcinoma (HCC) and hepatitis B virus-related liver cirrhosis and underwent CEUS-CT/MRI FI-guided radiofrequency ablation (RFA) with the use of SonoVue as a contrast agent. Arterial phase (**A**) and its subtraction MRI (**B**) demonstrates a small arterial-enhancing HCC (arrow) in liver segment 6. (**C**) In the hepatobiliary phase (20-min delayed phase), the tumor at segment 6 of the liver showed a clear defect (arrow). On B-mode US (**D**) and following fusion imaging (**E**), it was not possible to identify the HCC on B-mode US at the corresponding site with fused MRI (arrow). Therefore, the index tumor was assigned a conspicuity score of 1, since it was definitely unidentifiable on fusion imaging. (**F**) Arterial-phase imaging obtained post-SonoVue injection showed a small enhanced lesion (arrow) that could be clearly identified at the corresponding location on fused MRI. (**G**) Radiofrequency electrodes (arrowheads) were inserted into the index tumor (arrow) with CEUS-added fusion imaging guidance. (**H**) Portal-phase CT obtained immediately post-RFA demonstrates technical success and sufficient ablation margins (arrowheads). LTP was not identified at the site of ablation after 10 months of the procedure

**Fig. 3:**
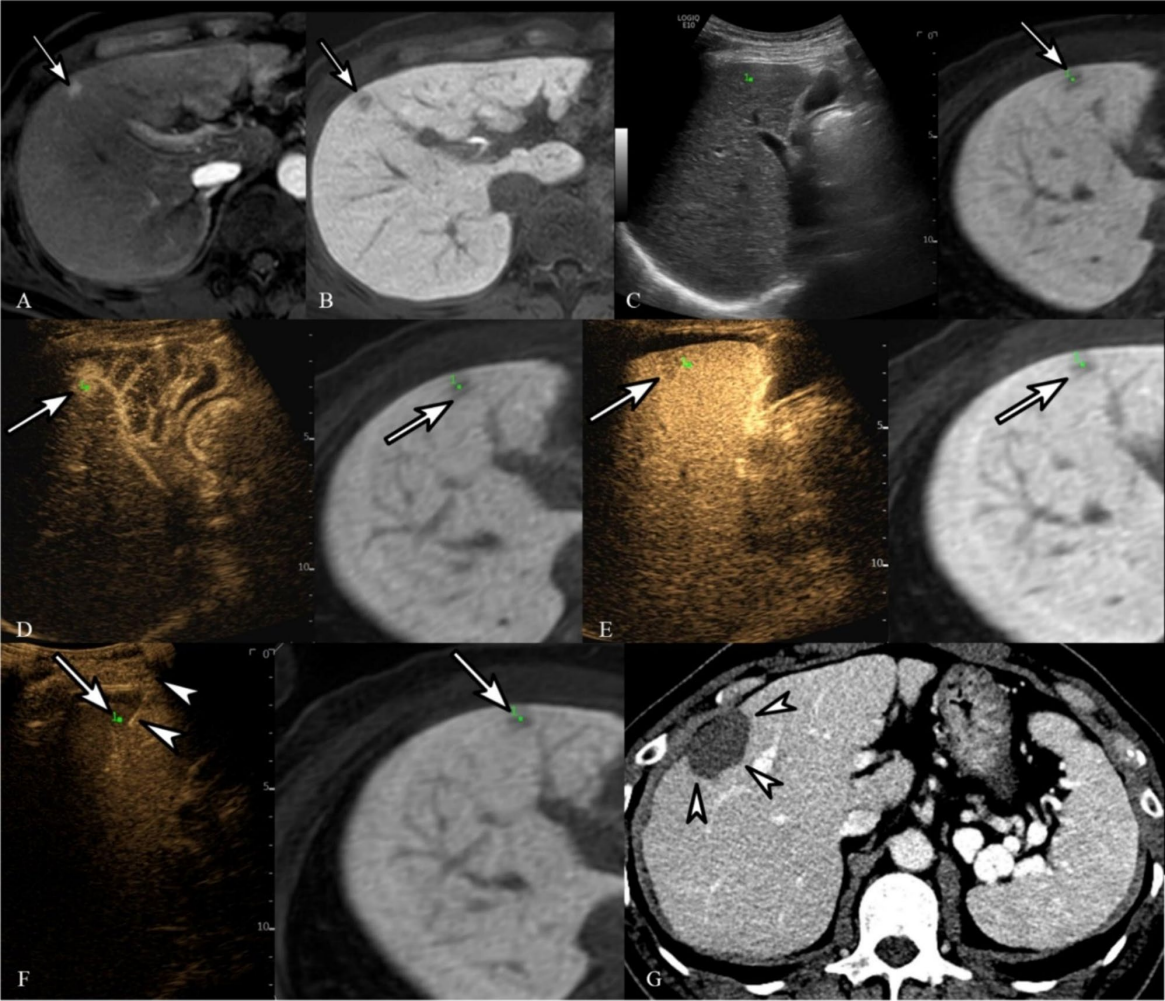
Images of using Sonazoid in radiofrequency ablation. Images of a 58-year-old man who had a 1.1-cm hepatocellular carcinoma (HCC) and hepatitis B virus-related liver cirrhosis and underwent CEUS-CT/MRI FI-guided radiofrequency ablation (RFA) with the use of Sonazoid as a contrast agent. (**A**) Arterial-phase MRI demonstrates a small hypervascular HCC (arrow) at the border of liver segments 5 and 8. (**B**) In hepatobiliary-phase (20-min delayed phase) MRI, the tumor at segment 5/8 border of the liver showed an apparent defect (arrow). (**C**) Following fusion imaging, it was not possible to identify the HCC on B-mode US imaging at the corresponding site on fused MRI (arrow). Therefore, the index tumor was assigned a conspicuity score of 1, as it was unidentifiable on fusion imaging. (**D**) Arterial-phase imaging obtained post-Sonazoid injection demonstrated an identifiable small enhanced lesion (arrow) at the corresponding location on fused MRI. (**E**) Kupffer-phase imaging identified the HCC (arrow) at the corresponding location on fused MRI. Therefore, the index tumor was assigned a conspicuity score of 4 on CEUS-added fusion imaging. (**F**) Radiofrequency electrodes (arrowheads) were positioned in the index tumor (arrow) with CEUS-CT/MRI FI guidance. (**G**) LTP was not identified at the site of ablation on portal-phase CT obtained 17 months post-RFA (arrowheads)

As patients with only inconspicuous or invisible tumors on B-mode US were enrolled, the proportion of conspicuous tumors significantly increased after adding CEUS-CT/MRI FI (88.7%, 220/248) (Table 3). Furthermore, the tumor visibility score of the total population significantly increased after using CEUS-CT/MRI FI compared with US-FI (1.9 ± 0.6 vs. 3.4 ± 0.7, *P* < 0.001) (Table 4). In addition, the expected technical feasibility score for the total population significantly increased after using CEUS-CT/MRI FI (2.9 ± 0.8 vs. 3.6 ± 0.6, *P* < 0.001). The tumor visibility and technical feasibility scores significantly increased after using CEUS-CT/MRI FI, regardless of tumor location and size or US contrast agents (Table 3). Both SonoVue- (Fig. 2) and Sonazoid-enhanced US-FI (Fig. 3) provided improved visibility higher or equal to grade 3 in 87.7% (121/138) and 90% (99/110) of tumors, respectively (*P* < 0.001).

**Table 3:** Visibility and feasibility results of CEUS-CT/MR FI for inconspicuous tumors

	Visibility score 3 or 4	Feasibility score 3 or 4	p-value
Total	220/248 (88.7)	235/248 (94.8)	< 0.001
**By location (binominal)**			< 0.001
Left lobe	57/62 (91.9)	59/62 (95.2)	
Right superior segments	84/99 (84.8)	91/99 (91.9)	
Right inferior segments	79/87 (90.8)	85/87 (97.7)	
**By size (binominal)**			< 0.001
< 2 cm	142/168 (84.5)	156/168 (92.9)	
≥ 2 cm, < 2.5 cm	62/64 (96.9)	63/64 (98.4)	
≥ 2.5 cm	16/16 (100)	16/16 (100)	
**By contrast agent (binominal)**			< 0.001
SonoVue	121/138 (87.7)	130/138 (94.2)	
Sonazoid	99/110 (90.0)	105/110 (95.5)	

*Note*: Unless indicated, data are number of tumors, with the percentage in parentheses

**Table 4:** Scores for tumor visibility and expected technical feasibility on US-FI and CEUS-CT/MR FI

	Tumor visibility			Technical feasibility		
	US-FI	CEUS-CT/MR FI	P value	US-FI	CEUS-CT/MR FI	P value
Total	1.9 ± 0.6	3.4 ± 0.7	< 0.001	2.9 ± 0.8	3.6 ± 0.6	< 0.001
**By location**						
Left lobe	2.0 ± 0.7	3.5 ± 0.7	< 0.001	2.8 ± 0.8	3.5 ± 0.6	< 0.001
Right superior segments	1.9 ± 0.6	3.3 ± 0.8	< 0.001	2.8 ± 0.8	3.5 ± 0.7	< 0.001
Right inferior segments	1.9 ± 0.6	3.4 ± 0.7	< 0.001	3.0 ± 0.8	3.8 ± 0.5	< 0.001
**By size**						
< 2 cm	1.8 ± 0.6	3.3 ± 0.8	< 0.001	2.8 ± 0.9	3.6 ± 0.7	< 0.001
≥ 2 cm, < 2.5 cm	2.0 ± 0.5	3.6 ± 0.5	< 0.001	3.0 ± 0.7	3.7 ± 0.5	< 0.001
≥ 2.5 cm	2.4 ± 0.5	3.6 ± 0.5	< 0.001	3.3 ± 0.6	3.7 ± 0.5	0.014
**By contrast agent**						
SonoVue	1.9 ± 0.7	3.4 ± 0.8	< 0.001	2.9 ± 0.9	3.7 ± 0.6	< 0.001
Sonazoid	1.8 ± 0.5	3.4 ± 0.7	< 0.001	2.8 ± 0.8	3.6 ± 0.6	< 0.001

*Note*: US-FI = ultrasound-CT/MR fusion imaging, CEUS-CT/MR FI = contrast-enhanced US-CT/MR fusion imaging

In addition, in groups A and B, the Kupffer phase of Sonazoid US lasted a long time (after 5 to 20 min) and allowed for the identification of target tumors and guiding electrode placement on CEUS-CT/MRI FI, which was not possible with the shorter-lived Sonovue. Furthermore, the long periods of enhancement of Sonazoid during the Kupffer phase also allowed for improving registration between CEUS and CT/MR using a point-to-point registration of the hypoenhancing tumor and the index tumor on CT or MRI. Instead, when SonoVue was used, an electronic “virtual” target of real-time fusion US-MR/CT was used for tumor localization and guiding electrode placement.

### Therapeutic outcomes of CEUS-assisted RFA procedures

The technical success rate was 99.6% (247/248), and technique efficacy rate was 99.6% (247/248). One patient in whom technical success was not achieved underwent transcatheter arterial chemoembolization for the residual tumor the day after the RFA procedure. No major complications occurred. However, minor complications occurred in nine patients (3.6%, 9/248): occlusion of the subsegmental portal vein or hepatic vein (< 3 mm in diameter) (n = 5), mild thermal injury in the gallbladder (n = 3), and minimal contrast extravasation at the needling site on immediate follow-up CT in one patient, which spontaneously stopped without embolization.

**Fig. 4:**
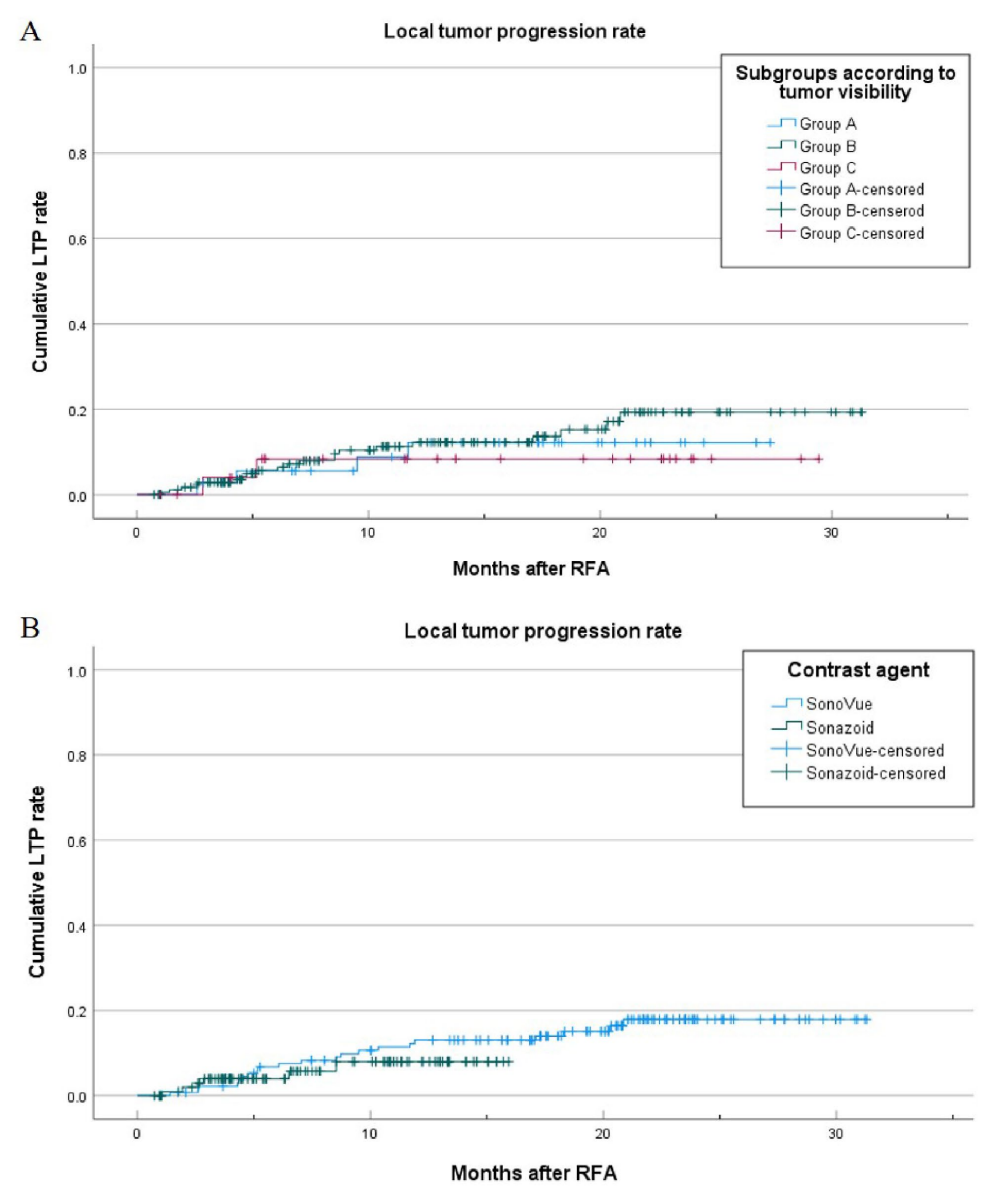
The Kaplan-Meier curve of the local tumor progression (LTP) rate after radiofrequency ablation. (**A**) LTP rate according to the subgroups divided by tumor visibility. (**B**) LTP rate according to the contrast agent used

During follow-up, the LTP rates at 1 and 2 years after CEUS-CT/MRI FI guided RFA were 9.3% (23/248) and 10.9% (27/248), respectively. The 1-year cumulative LTP rates of groups A, B, and C were 10.5% (4/38), 11.5% (21/183), and 7.4% (2/27), respectively (*P* > 0.05) (Fig. 4). The 1-year cumulative LTP rates in the SonoVue and Sonazoid groups were 12.3% (17/138) and 5.5% (6/110), respectively (Fig. 4). There was no statistically significant difference (*P* = 0.67).

## Discussion

This prospective study underscored the efficacy of CEUS-CT/MRI FI in enhancing both the visibility and feasibility of RFA for 85.5% of participants presenting with tumors indistinct on B-mode US. When considering contrast agents, CEUS-CT/MRI FI elevated the visibility to a grade 3 or higher in 87.7% of patients using SonoVue and 90% with Sonazoid, thereby optimizing the delineation of tumor margins for RFA. In terms of therapeutic metrics, CEUS-CT/MRI FI–guided RFA manifested commendable outcomes, evidenced by a 99.6% rate of technical success and technique efficacy, an absence of major complications, and a 10.9% rate of LTP over two years. It is noteworthy that our therapeutic outcomes for tumors elusive on B-mode US paralleled findings from our center’s prior research on visible tumors [28, 29]. This aligns with and reinforces prior literature, underscoring the clinical viability of CEUS-FI in directing RFA for tumors not readily discernible on standard US or US-FI [16, 18, 19]. Notably, our study’s cohort size surpassed those of earlier studies and incorporated two distinct US contrast mediums. Consequently, our findings offer robust evidence for the therapeutic potency of CEUS-CT/MRI FI-guided RFA when addressing small liver malignancies that are elusive on both B-mode and US-CT/MRI FI.

In this study, CEUS-CT/MRI FI significantly enhanced tumor visibility in 88.7% (220/248) of tumors that were indistinct on B-mode US, while US-FI elevated visibility for merely 26.2%. Numerous studies have underscored the efficacy of US-CT/MRI FI in addressing elusive tumors on B-mode US, enhancing either tumor visibility or the operator’s confidence in tumor localization [12, 13, 15]. However, while there might be heightened operator confidence for challenging tumors by co-localizing with pre-acquired CT/MRI data, the actual tumor visibility on US-CT/MRI FI might not always surpass that of standalone US [15]. Especially when tumors are entirely isoechoic on US, operators find themselves estimating the index tumor location based on a virtual target formulated through US-CT/MRI co-registration [16]. Notably, the inherent registration discrepancies between real-time US and pre-acquired CT/MRI can manifest in US-CT/MRI FI, especially when applying rigid registration to a deformable organ amidst movement. Such discrepancies raise concerns for incomplete ablation during FI-guided RFA [13, 15–17]. Our findings suggest that CEUS augments direct tumor visibility compared to traditional US, thereby enhancing the registration precision between the imaging modalities. Consequently, the virtual tumor marker on US images derived from CEUS-CT/MRI FI can be instrumental in electrode placement around the index tumor and the monitoring process. In essence, CEUS-CT/MRI FI offers indispensable support for ablation procedures, potentially circumventing technical challenges inherent to sole reliance on B-mode US or US-FI guidance.

Interestingly, CEUS-CT/MRI FI failed to localize the index tumor for ablation in 10.9% of tumors. Of these, a significant 92.6% were tumors smaller than 2 cm, with 55.6% predominantly located in the liver’s right superior segments. This observation aligns with a previous study which noted enhanced visualization using CEUS-FI for 90.5% of tumors that were otherwise indistinct on US-FI [16]. Such findings suggest that tumor size and anatomical positioning play a pivotal role in tumor conspicuity, even when augmented by CEUS. Previous studies also reported the difficulty of CEUS in assessing a deep-seated tumor greater than 10 cm from the transducer and tumors located superiorly in the dome of the liver. Furthermore, a significant fraction, approximately two-thirds, of early-stage HCCs with diameters less than 2 cm have been found to lack arterial enhancement on Sonazoid-CEUS [30, 31]. To circumvent these challenges, artificial ascites or pleural effusion, might optimize the sonic window for CEUS-FI. This technique seems particularly beneficial when addressing tumors located in the right superior segment, especially in areas beneath the diaphragm [28, 29].

Our study uniquely incorporated two predominant US contrast agents, SonoVue and Sonazoid. Regardless of the contrast medium employed, we observed marked improvements in tumor visibility and technical feasibility scores when utilizing CEUS-CT/MRI FI, as compared to US-FI (Table 4). A direct comparison of the efficacy between these two contrast agents proved challenging due to the non-randomized nature of our study. However, an operator-driven observation highlighted the superiority of the Kupffer-phase imaging under Sonazoid-enhanced US, especially when multiple electrodes were involved. This was primarily attributed to the elevated lesion contrast observed in the Kupffer-phase and the extended enhancement duration of liver parenchyma with Sonazoid [32, 33]. Sonazoid bubbles are notably more resilient than SonoVue due to distinct pharmacophysical attributes. Additionally, Sonazoid bubbles, once engulfed by Kupffer cells, exhibit a prolonged lifespan exceeding 10 min [33, 34]. Technical success, intriguingly, was almost at par for both agents – achieving 100.0% (138/138) with SonoVue and 99.1% (109/110) with Sonazoid. A sole case of incomplete procedure was observed with Sonazoid, stemming from a compromised sonic window due to extensive pleural calcification. Interestingly, despite the evident benefits of Sonazoid’s Kupffer phase, the 1-year cumulative LTP rates of CEUS-FI with Sonazoid (5.5%) and SonoVue (12.3%) showed no statistically significant difference. The main reason why the advantages of the Kupffer phase of Sonazoid-US failed to deliver a better therapeutic outcome of RFA compared with SonoVue-enhanced US could be related to the use of an electronic “virtual” target of real-time fusion CEUS-MR/CT for tumor localization and guiding electrode placement. Randomized controlled trials comparing the therapeutic efficacy of SonoVue- and Sonazoid-enhanced US FI for guiding RFA are necessary.

This study had several limitations. First, being a single-center study conducted at a tertiary academic hospital in an East Asian country potentially limits the generalizability of our results. However, our study also has notable strengths, including the enrollment of a large number of patients and the inclusion of both commercially available contrast agents for Contrast-Enhanced Ultrasound (CEUS). These factors contribute to the robustness and applicability of our findings. Second, 71.4% of patients had hepatitis B virus-related liver cirrhosis, which may hinder generalization of the results to other settings where hepatitis B virus is uncommon. In general, hepatitis B viral infection-induced liver cirrhosis leads to the development of macronodular cirrhosis with prominent regenerative or dysplastic nodules, making it challenging to localize HCCs on planning US [35]. Third, we did not evaluate patients’ overall survival. However, the primary goal of this study was to assess the value of CEUS-CT/MRI FI in performing percutaneous RFA of small malignant liver tumors that were inconspicuous on B-mode US. Last, in terms of contrast agents used in CEUS, Kupffer-phase imaging of Sonazoid-enhanced US was regarded as being more advantageous for placing multiple electrodes than the delayed phase of SonoVue-enhanced US. However, as this study was not a randomized controlled trial, comparing the results between SonoVue and Sonazoid may yield limited insights and be subject to potential selection bias.

## Conclusions

In conclusion, CEUS-CT/MRI FI substantially increased the feasibility of ablation for inconspicuous tumors on B-mode US by improving tumor visibility, leading to successful percutaneous RFA with excellent therapeutic outcomes.

### Electronic supplementary material

Below is the link to the electronic supplementary material.

**Supplementary Material 1:** Score categories for evaluation of target tumor visibility, technical feasibility and route safety

## Data Availability

Not applicable.

## List of abbreviations

CEUS: Contrast-enhanced ultrasound
CT: Computed tomography
FI: Fusion imaging
HCC: Hepatocellular carcinoma
LTP: Local tumor progression
MRI: Magnetic resonance imaging
RFA: Radiofrequency ablation
US: Ultrasound
