# Supplementary material for: Contrast-enhanced ultrasonography–CT/MRI fusion guidance for percutaneous ablation of inconspicuous, small liver tumors: improving feasibility and therapeutic outcome
Source: Cancer Imaging. 2024 Jan 3;24:4. doi: 10.1186/s40644-023-00650-y (PMC10762814; doi:10.1186/s40644-023-00650-y)
Supplement: Supplementary file 1 — Supplementary Material 1: Score categories for evaluation of target tumor visibility, technical feasibility and route safety [file 40644_2023_650_MOESM1_ESM.docx]

**SUPPLEMENTARY TABLES**

**Supplementary Table 1. Score categories for evaluation of target tumor visibility, technical feasibility and route safety**

| Definitions | |
| --- | --- |
| Target tumor visibility and localization confidence |  |
| 1 | Definitely invisible |
| 2 | Subtle visualization of the tumor with slightly different echogenicity compared with surrounding parenchyma and unclear tumor border |
| 3 | Visible tumor having slightly different echogenicity compared with surrounding parenchyma with partial delineation of tumor margin (50%~90%) |
| 4 | Clearly visible tumor having different echogenicity compared with surrounding parenchyma with a distinct margin ( > 90% of tumor border) |
| Technical feasibility |  |
| 1 | Low; invisible tumor, or poor safe access route |
| 2 | Moderate; partially visible tumor or tumor with poor conspicuity and a fair safe access route |
| 3 | High; fair tumor conspicuity and a fair safe access route |
| 4 | Highest; confident in identifying the index tumor and presence of a good safe access route |
| Route safety |  |
| 1 | Bad with segmental branches of PV or HV |
| 2 | Adequate with subsegmental small PV, HV branches |
| 3 | Safe route with no large vessels |
